# A Therapeutic DNA Vaccine Targeting HPV16 E7 in Combination with Anti-PD-1/PD-L1 Enhanced Tumor Regression and Cytotoxic Immune Responses

**DOI:** 10.3390/ijms242015469

**Published:** 2023-10-23

**Authors:** Xuechao Han, Zhixiao Gao, Yeping Cheng, Shuoshuo Wu, Jianxing Chen, Weifang Zhang

**Affiliations:** Department of Microbiology, School of Basic Medical Science, Cheeloo College of Medicine, Shandong University, Jinan 250012, China; xuechaohan001@163.com (X.H.); 15153866197@163.com (Z.G.); luhandwrg@163.com (Y.C.); ss101399@163.com (S.W.); jxchen@sdu.edu.cn (J.C.)

**Keywords:** human papillomavirus, cervical cancer, E7, therapeutic vaccine, PD-1

## Abstract

Persistent infection of high-risk human papillomavirus (HPV) and the expression of E6 and E7 oncoproteins are the main causes of cervical cancer. Several prophylactic HPV vaccines are used in the clinic, but these vaccines have limited efficacy in patients already infected with HPV. Since HPV E7 is vital for tumor-specific immunity, developing a vaccine against HPV E7 is an attractive strategy for cervical cancer treatment. Here, we constructed an HPV16 E7 mutant that loses the ability to bind pRb while still eliciting a robust immune response. In order to build a therapeutic DNA vaccine, the E7 mutant was packaged in an adenovirus vector (Ad-E7) for efficient expression and enhanced immunogenicity of the vaccine. Our results showed that the Ad-E7 vaccine effectively inhibited tumor growth and increased the proportion of interferon-gamma (IFN-γ)-secreting CD8^+^ T cells in the spleen, and tumor-infiltrating lymphocytes in a mouse cervical cancer model was achieved by injecting with HPV16-E6/E7-expressing TC-1 cells subcutaneously. Combining the Ad-E7 vaccine with the PD-1/PD-L1 antibody blockade significantly improved the control of TC-1 tumors. Combination therapy elicited stronger cytotoxic T lymphocyte (CTL) responses, and IFN-γ secretion downregulated the proportion of Tregs and MDSCs significantly. The expressions of cancer-promoting factors, such as TNF-α, were also significantly down-regulated in the case of combination therapy. In addition, combination therapy inhibited the number of capillaries in tumor tissues and increased the thickness of the tumor capsule. Thus, Ad-E7 vaccination, in combination with an immune checkpoint blockade, may benefit patients with HPV16-associated cervical cancer.

## 1. Introduction

Cancer is a major cause of death, and the incidence rate and mortality of cervical cancer (CC) is high for women worldwide [1]. Persistent high-risk human papillomavirus (HR-HPV) infection plays a causal role in the development of CC [2]. HPV genome encodes six early proteins—E1, E2, E4, E5, E6 and E7—and two late structural proteins, L1 and L2. The major capsid protein of HPV L1 assembles into virus-like particles (VLPs), which are morphologically and antigenically similar to HPV virions and are regarded as the prophylactic vaccine of HPV [3]. The FDA-approved prophylactic HPV vaccines include a bivalent (HPV16, 18), quadrivalent (HPV6, 11, 16, 18) and 9-valent vaccine, which covers HPV types 6, 11, 16, 18, 31, 33, 45, 52, and 58 [4]. These vaccines protect against HPV-related cancers such as cancers of the cervix, vulva, vagina, penis, anus, and oropharynges, as well as a high-grade cervical intraepithelial neoplasia (CIN), mainly by the induction of a high-titer of durable, neutralizing antibody responses, which block the entrance of viral particles into host cells [5]. However, only 7.5% of women worldwide have received the HPV vaccine at the age of 10–20 [6]. Additionally, vaccination has limited efficacy in patients already infected with HPV since L1 is not expressed in infected epithelial cells [7]. Therefore, therapeutic vaccines are urgently required.

HPV oncogenes E6 and E7 are integrated into host chromosomal DNA, essential for cellular transformation and tumor development [8]. Early oncoprotein E6 induces degradation of the host tumor suppressor protein p53 [9], while E7 binds to and degrades the retinoblastoma protein (pRb), resulting in the release of transcription factor E2F from the pRb-E2F complex and leading to S phase entry and genomic instability [10]. Since HPV E6 and E7 are expressed constitutively, they become ideal targets for HPV therapeutic vaccines that could induce robust tumor-specific T-cell type 1 and cytotoxic T lymphocyte (CTL) responses to kill HPV-infected cells [11], of which E7 represents the most attractive target for antigen-specific immunity of cervical cancer. Several E7-based vaccines have been developed, which include viral vector-based or bacterial vector-based, nucleic acid-based (DNA and RNA), peptide-/protein-based, or cell-based vaccines [12]. Among these vaccines, DNA vaccines offer a stable, safe, and simple manufacturing process, making them a potentially effective approach to treat HPV-associated cancers [13]. For example, the HPV-16 E7 mutant E7GGG (E7 without Rb binding site), which was expressed via the recombinant tobacco mosaic virus, had strong humoral and immune responses and inhibited tumor growth [14,15]. However, DNA vaccination with HPV E7 is poorly immunogenic, and co-administration of immunotherapy is needed to elicit strong immune responses.

Programmed death-1 (PD-1) is an immune checkpoint receptor expressed on the surface of CTLs, which interacts with programmed death-ligand 1 (PD-L1) on antigen-presenting cells (APCs), resulting in the attenuation of CTL activation [16]. This immune checkpoint pathway is used by ‘self’ cells to prevent autoimmunity, but tumor cells can hijack this mechanism to escape from immunosurveillance [17]. Blocking this immune checkpoint signaling helps overcome immunosuppression. Inhibitors targeted at PD-1 or PD-L1 restore the function of CTLs, thereby enhancing the natural anti-tumor immune responses. PD-1 and PD-L1 antibodies have been demonstrated to be potent in treating several types of cancers, including cutaneous melanoma, non-small cell lung cancer, renal cell carcinoma, bladder cancer, head and neck cancers, as well as Hodgkin’s lymphoma [18]. Additionally, PD-1 and PD-L1 are widely expressed in cervical cancer tumor cells and the application of a PD-1 antibody improves prognosis for a fraction of cervical cancer patients [19].

In this study, an adenoviral vector encoding a mutant HPV16 E7 (delD21–C24) was developed (Ad-E7). The mutations of E7 were the deletion of amino acids 21–24, resulting in the loss of the capability to bind to pRb and transformation activity. The positions of the point mutations are different from previous studies [20,21]. The high titre of neutralizing antibodies in humans against adenovirus serotype 5 (Ad5) gives the Ad-E7 vaccine a more unique advantage [22]. We demonstrated that Ad-E7 was an effective candidate vaccine, and upon combination with PD-1/PD-L1 antibody, induced a strong immune response and inhibited tumor growth in mouse cervical cancer models. Our results showed that the persistent expression of the Ad-E7 protein robustly increased specific CD8^+^ T cell responses and therapeutic effects. In addition, the PD-1/PD-L1 antibody blocked the signaling between tumor cells and immune-related regulatory cells and T cells, and prevented tumor cells from evading immune surveillance. Therefore, combining PD-1/PD-L1 antibody treatment with Ad-E7 vaccination may be a promising therapeutic option for HPV16-associated cervical cancer.

## 2. Results

### 2.1. Production and Identification of Ad-E7 Adenovirus Vaccine

**Figure 1:**
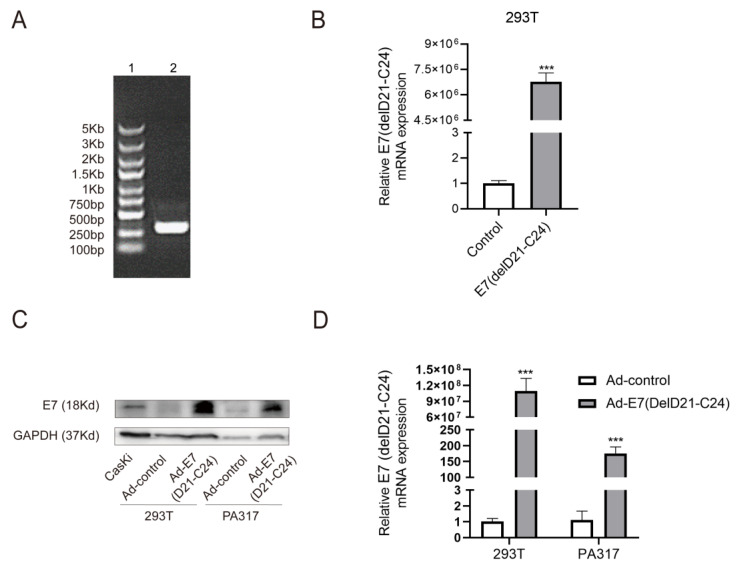
Construction and identification of the Ad-E7 vaccine. (**A**) The amplified E7 (delD21-C24) gene was identified using agarose gel electrophoresis. Lane 1 is the DNA marker, which is 5 Kb, 3 Kb, 2 Kb, 1.5 Kb, 1 Kb, 750 bp, 500 bp, 250 bp and 100 bp from top to bottom, and lane 2 is E7 (delD21-C24). (**B**) E7 (delD21-C24) mRNA level was detected using qRT-PCR following transfection of E7 (delD21-C24) plasmid into 293T cells. (**C**) Western blot analysis of E7 (delD21-C24) protein level in the 293T and PA317 cells infected by Ad-E7 adenovirus in vitro. (**D**) qRT-PCR was employed to detect mRNA level of E7 (deld21-c24) in the 293T and PA317 cells infected by Ad-E7 adenovirus in vitro. *** *p* < 0.001.

The HPV16 E7 gene contains 297 nucleotides and encodes 98 amino acids. The HPV16 E7 mutant (delD21-C24) [23], which loses the ability to interact with pRb, contains 285 nucleotides and encodes 94 amino acid sequences (Figure S1A). We first synthesized the E7 mutant, primarily verified via agarose gel electrophoresis (Figure 1A).

The GV269 vector was digested by Age I and EcoR I, and subsequently verified by agarose gel electrophoresis (Figure S1B). To generate the mutant E7 plasmid, homologous recombination was carried out between the E7 (delD21-C24) fragment and the digested-GV269 vector and then transformed into competent cells, culturing in an LB medium at 37 °C overnight. Eight monoclonal colonies were selected to amplify the E7 (delD21-C24) gene and were further detected using agarose gel electrophoresis. The results proved the correctness of positive transformants that were screened, except lanes 8 and 12 (Figure S1C). One of the positive transformants, named ‘sample 1’, was selected for sequencing analysis, and the sequencing results showed that the gene sequence of ‘sample 1’ was E7 (delD21-C24). Agarose gel electrophoresis further proved the construction of the E7 mutant. To evaluate the expression of E7 (delD21-C24), we extracted the mutant E7 plasmid and transfected it into 293T cells. The results showed that the mRNA level of E7 (delD21-C24) was significantly increased compared to the control (Figure 1B).

To obtain the recombined adenovirus Ad-E7, the mutant E7 plasmid and a large backbone plasmid, which contains most of the adenovirus genome, were co-transfected into HEK293 cells, and then a Cre-loxP (or FLP/frt) recombinase cleavage system was used for virus amplification. After two rounds of amplification, the virus titer was about 3 × 10^10^ PFU/mL. In order to verify the effectiveness of the vaccine, PA317 cells and 293T cells were used for infection experiments in vitro. The cervical cancer cell line, CasKi (HPV-16 positive), which expresses E7, was used as a positive control. The results showed the successful expression of the E7 (delD21-c24) protein and mRNA in these cells (Figure 1C,D).

### 2.2. Ad-E7 Combined with PD-1/PD-L1 Antibody Inhibits Tumor Growth in Mice

In order to verify whether TC-1 cells express HPV16 E7, we used PA317 cells and CasKi cells as negative and positive controls, respectively. The expression of the HPV16 E7 protein and mRNA in TC-1 cells was confirmed (Figure S2A,B). To directly compare the impact of different cell concentrations on the growth kinetics of tumors, we inoculated mice with 1 × 10^6^, 5 × 10^5^, or 2 × 10^5^/100 μL TC-1 cells to establish tumors at the left shaved flank. Different concentrations of TC-1 cells generated visible subcutaneous tumors (Figure S2C,D). Considering the duration of effectiveness of the vaccination, the time of observation, and the requirements of animal ethics, we selected a cell concentration of 2 × 10^5^/100 μL for the following experiments.

**Figure 2:**
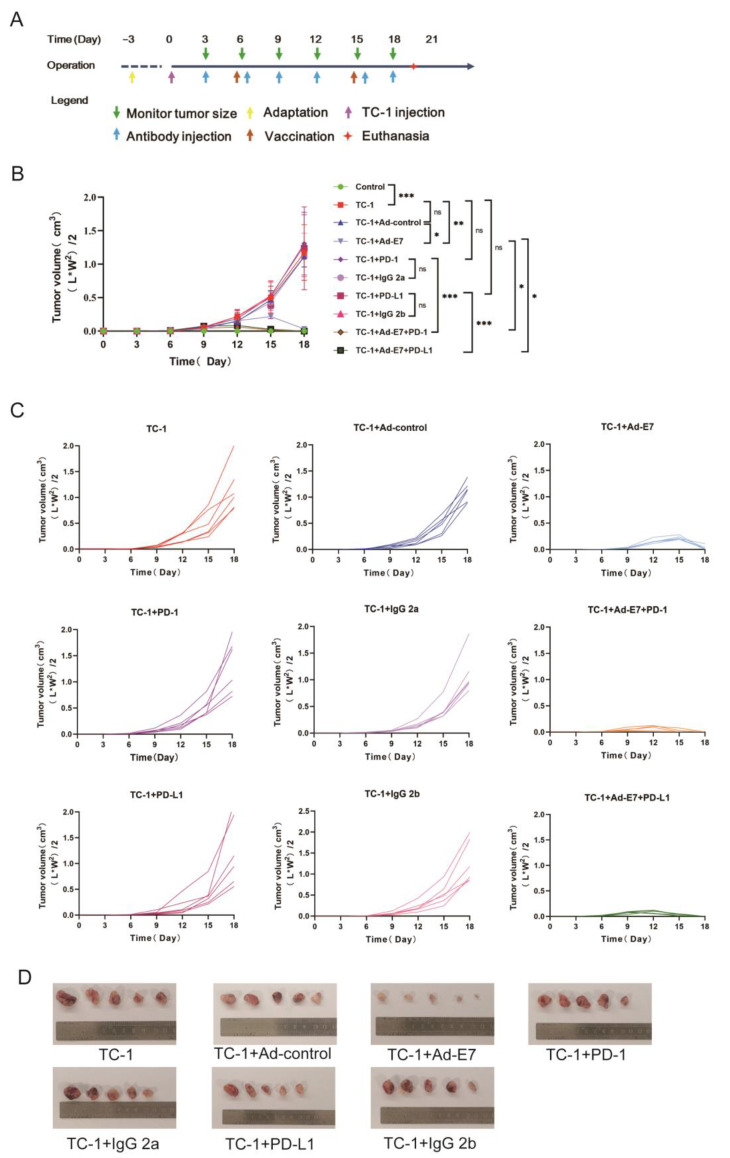
Effects of different treatments on tumor growth in tumor models. (**A**) Schematic diagram of treatment time in tumor-bearing mice. Purple arrow indicates the time points for injection of TC-1 cells; green arrows indicate the time points for measurement of tumors every three days; blue arrows represent the time points for injection of related antibodies using the indicated dosage; brown arrows represent the time points for vaccination at day 6 and day 15 (for booster immunization). (**B**) Average tumor growth curve of mice in each group. (**C**) Tumor growth curve of each mouse in each group with different treatments (n = 6/group). (**D**) The tumors of the mice were isolated and photographed. * *p* < 0.05, ** *p* < 0.01, *** *p* < 0.001, ns = not statistically significant (*p* > 0.05).

TC-1 cells were injected into the left flank of C57BL/6 mice. After 6 days, mice were divided into ten groups according to stratified randomization based on tumor size to make sure that each group has similar average tumor size, and each group of mice was given a different treatment (group 1: control; group 2: TC-1; group 3: TC-1 + Ad-control; group 4: TC-1 + Ad-E7; group 5: TC-1 + PD-1 antibody; group 6: TC-1 + IgG 2a; group 7: TC-1 + PD-L1 antibody; group 8: TC-1 + IgG 2b; group 9: TC-1 + Ad-E7 + PD-1 antibody; group 10: TC-1 + Ad-E7 + PD-L1 antibody). The time diagram is shown in Figure 2A. Tumors were measured and recorded every 3 days to draw the growth curve of the tumor. It was shown that there were no significant differences in group 3 (TC-1 + Ad-control), group 5 (TC-1 + PD-1 antibody), and group 7 (TC-1 + PD-L1 antibody), compared with group 2 (TC-1) (Figure 2B,C). However, the Ad-E7 vaccine suppressed tumor growth, and the tumors became smaller after day 15 and gradually subsided. Importantly, a more significant decrease in the size of the tumor was found in groups that received Ad-E7 along with PD-1 or PD-L1 antibody. In group 9 (TC-1 + Ad-E7 + PD-1 antibody) and group 10 (TC-1 + Ad-E7 + PD-L1 antibody), the tumors were scarcely detected after day 12. When the tumor volume reached 1.5 cm^3^ in group 2 (TC-1), all mice were euthanized, and tumors were dissected and photographed, except the tumors in group 9 (TC-1 + Ad-E7 + PD-1) and group 10 (TC-1 + Ad-E7 + PD-L1), which had disappeared (Figure 2D). These data demonstrate that Ad-E7 combined with a PD-1/PD-L1 antibody inhibits tumor growth.

### 2.3. Ad-E7 Combined with PD-1/PD-L1 Antibody Induces Strong CTL Responses in Spleen

Cell-mediated immune responses are more important for clearing established infections than humoral immune responses. Studies have shown that the cytotoxicity mediated by CD8^+^ T lymphocytes is crucial for the clearance of intracellular infections and malignant cells [24]. CD8^+^ T cells eliminate pathogens by directly killing infected cells and secreting cytokines, such as IFN-γ, which play a central role in anti-viral infection and anti-tumor immunity [25]. Tumor cells can promote tumor metastasis by regulating the recruitment and expansion of immunosuppressive cell population, for example, regulatory T cells (Tregs) and myeloid-derived suppressor cells (MDSCs), which promote the occurrence and development of cancer [26]. Consistently reducing the proportion of Tregs corrects the immune imbalance in suppressing or enhancing tumor immunity and consequently inhibits tumor growth [27].

**Figure 3:**
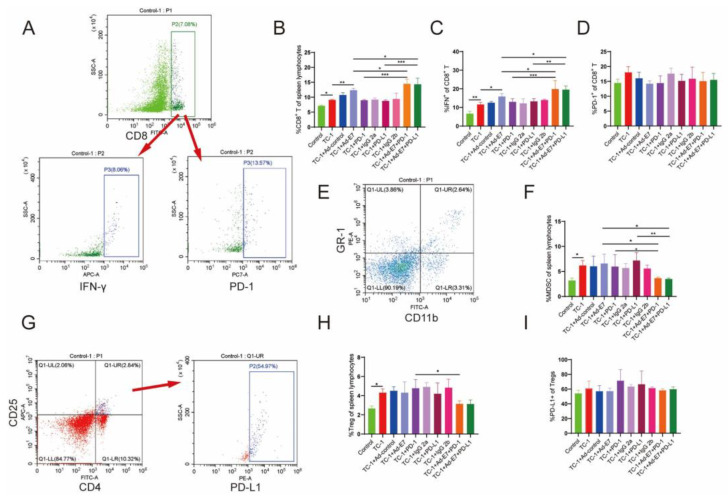
Expression of related immune cells in spleens of mice. (**A**) CD8^+^ T lymphocyte populations were analyzed using flow cytometry. (**B**) The frequencies of CD8^+^ T lymphocytes in spleen lymphocytes in each group with different treatments. (**C**) The positive proportion of IFN-γ in CD8^+^ T lymphocytes in each group with different treatments. (**D**) The positive proportion of PD-1 in CD8^+^ T lymphocytes in each group with different treatments. (**E**) MDSC populations were analyzed using flow cytometry. (**F**) The proportion of MDSCs in spleen lymphocytes in each group with different treatments. (**G**) Treg populations were detected using flow cytometry. (**H**) The proportion of Tregs in spleen lymphocytes in each group with different treatments. (**I**) The positive ratio of PD-L1 in Tregs in each group with different treatments. Bar graph is used to summarize the flow cytometry data. * *p* < 0.05, ** *p* < 0.01, *** *p* < 0.001.

We then detected the lymphocytes in the spleen of mice (Figure 3A). The results showed that TC-1 inoculation stimulated the production of CD8^+^ T lymphocytes, and the mice that received Ad-E7 had a marked increase in the frequency of CD8^+^ T cells (Figure 3B). Group 9 (TC-1 + Ad-E7 + PD-1) and group 10 (TC-1 + Ad-E7 + PD-L1) induced more CD8^+^ T cell responses compared to other groups (TC-1 + Ad-E7, TC-1 + PD-1, TC-1 + PD-L1). Ad-E7 moderately elevated the level of IFN-γ, while the combination of Ad-E7 and PD-1/PD-L1 antibody in group 9 (TC-1 + Ad-E7 + PD-1) and group 10 (TC-1 + Ad-E7 + PD-L1) displayed the highest induction of IFN-γ (Figure 3C). However, there were no significant differences in PD-1 expression of CD8^+^ T lymphocytes in all groups (Figure 3D).

MDSCs in the spleen of mice were then detected, and we showed that TC-1 injection stimulated the production of MDSCs, while Ad-E7 did not influence the proportion of MDSCs (Figure 3E,F). In addition, no significant differences were observed between group 5 (TC-1 + PD-1) or group 7 (TC-1 + PD-L1) and group 2 (TC-1). However, the combination of Ad-E7 and PD-1/PD-L1 antibody significantly decreased the proportion of MDSCs.

Tregs were analyzed simultaneously, and we showed that the proportion of Tregs was not decreased after the vaccination with Ad-E7 (Figure 3G,H). Similarly, there were no significant changes in the proportion of Tregs when treated with PD-1 or PD-L1 antibody alone (TC-1 + PD-1, TC-1 + PD-L1). However, the proportion of Tregs was decreased significantly in the combination therapy with the Ad-E7 and PD-1 antibody (TC-1 + Ad-E7 + PD-1 vs. TC-1 + PD-1). Further detection showed that there were no significant differences in PD-L1 expression of Tregs in all groups (Figure 3I).

### 2.4. Ad-E7 Combined with PD-1/PD-L1 Antibody Induces Strong CTL Responses in Tumors

**Figure 4:**
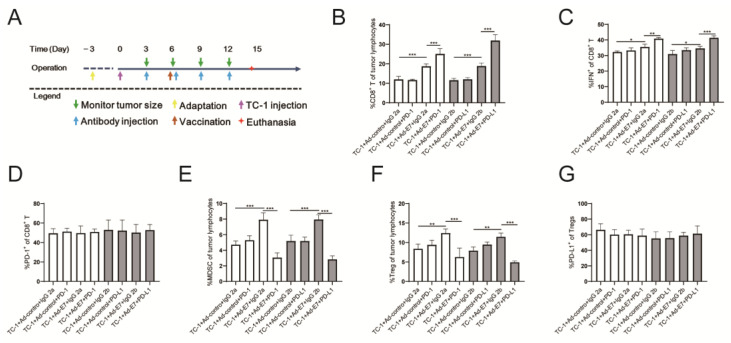
Analysis of tumor-infiltrating lymphocytes (TILs). (**A**) Schematic diagram of treatment time in tumor-bearing mice. Purple arrow indicates the time points for injection of TC-1cells; green arrows indicate the time points for measurement of tumors every three days; blue arrows represent the time points for injection of related antibodies using the indicated dosage; brown arrow represents the time points for vaccination at day 6. (**B**) The proportion of CD8^+^ T lymphocytes in TILs. The white bars represent the first part being the Ad-E7 vaccine combined with the PD-1 antibody, and the gray bars represent the second being the Ad-E7 vaccine combined with the PD-L1 antibody. (**C**) The positive proportion of IFN-γ in CD8^+^ T lymphocytes. (**D**) The positive proportion of PD-1 in CD8^+^ T lymphocytes. (**E**) The positive proportion of MDSCs in TILs. (**F**) The positive proportion of Tregs in TILs. (**G**) The positive proportion of PD-L1 in Tregs. Bar graph is used to summarize the flow cytometry data. * *p* < 0.05, ** *p* < 0.01, *** *p* < 0.001.

Since the tumors in group 9 (TC-1 + Ad-E7 + PD-1) and group 10 (TC-1 + Ad-E7 + PD-L1) had subsided after day 15 during the first inoculation, we could not detect the proportion of tumor-infiltrating lymphocytes (TILs). Therefore, we redesigned the experimental process to detect TILs (Figure 4A). The experiment consisted of two parts, the first part being the Ad-E7 vaccine combined with the PD-1 antibody (TC-1 + Ad-control + IgG 2a, TC-1 + Ad-control + PD-1, TC-1 + Ad-E7 + IgG 2a, and TC-1 + Ad-E7 + PD-1), and the second being the Ad-E7 vaccine combined with the PD-L1 antibody (TC-1 + Ad-control + IgG 2b, TC-1 + Ad-control + PD-L1, TC-1 + Ad-E7 + IgG 2b, and TC-1 + Ad-E7 + PD-L1). All mice were euthanized 14 days after injection, and tumor tissues were isolated to detect TILs.

We found that the proportion of CD8^+^ T lymphocytes in the tumors increased after vaccination with Ad-E7, and the proportion of CD8^+^ T lymphocytes in the Ad-E7 groups and combination groups (TC-1 + Ad-E7 + IgG 2a, TC-1 + Ad-E7 + PD-1, TC-1 + Ad-E7 + IgG 2b, and TC-1 + Ad-E7 + PD-L1) were higher than the corresponding control groups (TC-1 + Ad-control + IgG 2a, TC-1 + Ad-control + PD-1, TC-1 + Ad-control + IgG 2b, and TC-1 + Ad-control + PD-L1) (Figure 4B). Further analysis of CD8^+^ T lymphocytes showed the highest expression of IFN-γ in the TC-1 + Ad-E7 + PD-1 and TC-1 + Ad-E7 + PD-L1 groups (Figure 4C). There were no significant differences in PD-L1 expression of CD8^+^ T lymphocytes (Figure 4D). Furthermore, the proportion of MDSCs in infiltrating lymphocytes in the TC-1 + Ad-control + PD-1 and TC-1 + Ad-control + PD-L1 groups did not change significantly compared to the corresponding control groups (TC-1 + Ad-control + IgG 2a, TC-1 + Ad-control + IgG 2b), while the proportion of the MDSCs in the Ad-E7 groups (TC-1 + Ad-E7 + IgG 2a, TC-1 + Ad-E7 + IgG 2b) were up-regulated (Figure 4E). However, the combination therapy with Ad-E7 and PD-1/PD-L1 antibody significantly decreased the proportion of the MDSCs. Similar results were achieved with regard to the proportion of Tregs (Figure 4F). Detection of PD-L1 expression in Tregs cells showed no significant differences among all groups (Figure 4G).

### 2.5. Ad-E7 Combined with PD-1/PD-L1 Antibody Inhibits the Secretion of Cytokines in Tumors

The recruitment and expansion of immune cells in local tumors are closely related to cytokines and chemokines, such as CCL2 (C-C motif chemokine ligand 2), which is important in maintaining MDSC activity [28]. In addition, a variety of cytokines, including TNF-alpha (TNF-α), TGF-beta (TGF-β), MMP2 (matrix metalloproteinase-2), and MMP3, are associated with the proliferation, invasion and metastasis of tumor cells [29,30,31,32].

**Figure 5:**
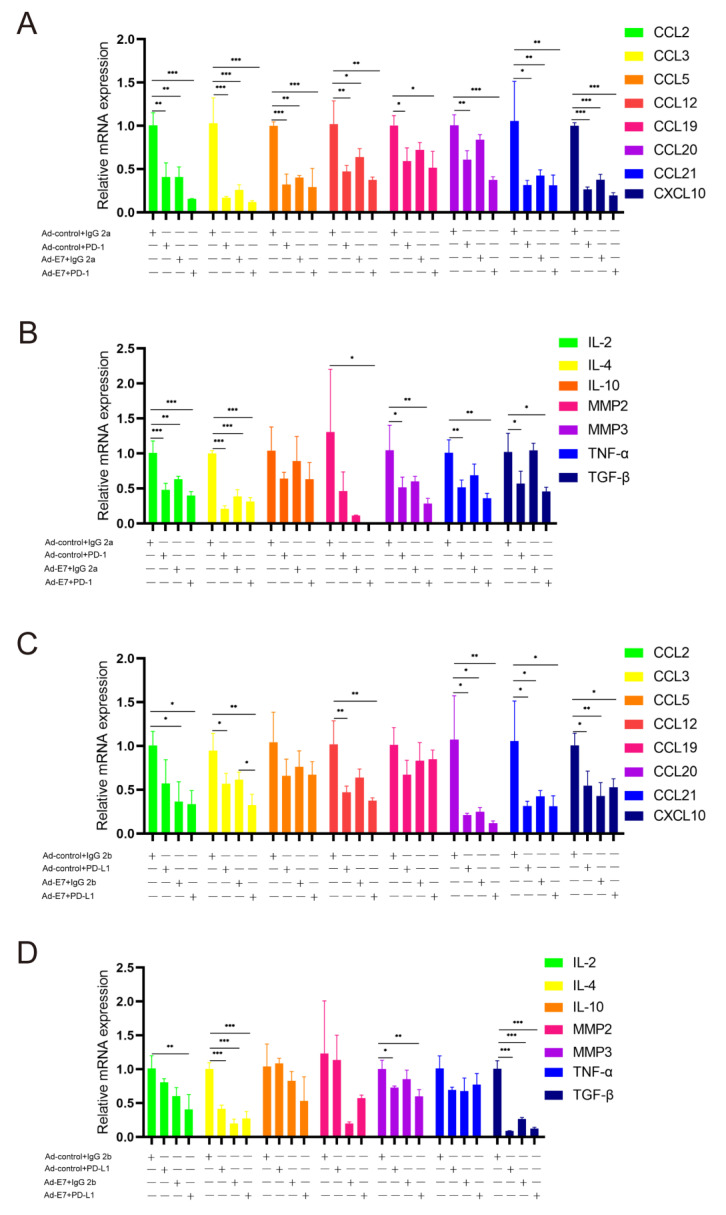
Expression of related cytokines in tumors. (**A**,**B**) qRT-PCR analysis of CCL2, CCL3, CCL5, CCL12, CCL19, CCL20, CCL21, CXCL10, IL-2, IL-4, IL-10, MMP2, MMP3, TNF-α, and TGF-β mRNA levels in four groups (TC-1 + Ad-control + IgG 2a, TC-1 + Ad-control + PD-1, TC-1 + Ad-E7 + IgG 2a, TC-1 + Ad-E7 + PD-1). (**C**,**D**) qRT-PCR analysis of CCL2, CCL3, CCL5, CCL12, CCL19, CCL20, CCL21, CXCL10, IL-2, IL-4, IL-10, MMP2, MMP3, TNF-α, and TGF-β mRNA levels in four groups (TC-1 + Ad-control + IgG 2b, TC-1 + Ad-control + PD-L1, TC-1 + Ad-E7 + IgG 2b, TC-1 + Ad-E7 + PD-L1). * *p* < 0.05, ** *p* < 0.01, *** *p* < 0.001.

The RNA of tumor tissues in each group was extracted and analyzed by the qRT-PCR assay. We detected the expression of related cytokines, including CCL2, CCL3, CCL5, CCL12, CCL19, CCL20, CCL21; CXCL10 (C-X-C motif chemokine); IL-2 (Interleukin-2), IL-4, IL-10; MMP2, MMP3; TNF-α; and TGF-β (Figure 5A–D). The results showed that, compared with the corresponding control groups (TC-1 + Ad-control + IgG 2a, TC-1 + Ad-control + IgG 2b), the expression of related cytokines in the four experimental groups (TC-1 + Ad-control + PD-1, TC-1 + Ad-control + PD-L1, TC-1 + Ad-E7 + IgG 2a, and TC-1 + Ad-E7 + IgG 2b) were decreased significantly. However, no significant difference was observed between combination therapy (TC-1 + Ad-E7 + PD-1, TC-1 + Ad-E7 + PD-L1) and monotherapy with the Ad-E7 or PD-1/PD-L1 antibody (TC-1 + Ad-E7 + IgG 2a, TC-1 + Ad-E7 + IgG 2b, TC-1 + Ad-control + PD-1, TC-1 + Ad-control + PD-L1) for CCL2.

### 2.6. Ad-E7 Combined with PD1/PD-L1 Antibody Decreases Tumor Angiogenesis and Increases Capsule Thickness

Histologically, a tumor capsule is a structure which can separate a tumor from the normal tissue. It has been traditionally considered as a sign of tumor benignity that prevent tumor cells’ local invasion and limit tumor cells spreading to other parts of the body to some extent [33]. The formation of new blood vessels is important to tumor growth and canceration, which facilitates the transportation of oxygen and nutrients to support tumor proliferation, invasion, and metastasis [34].

**Figure 6:**
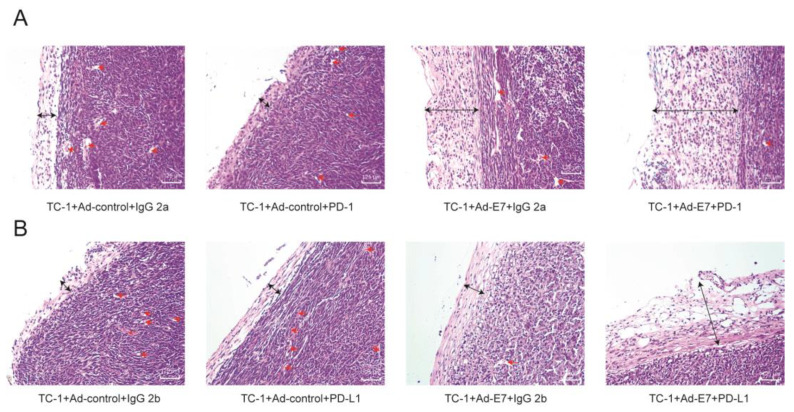
The H&E staining of tumor tissues. (**A**) The H&E staining of tumor tissues in four groups (TC-1 + Ad-control + IgG 2a, TC-1 + Ad-control + PD-1, TC-1 + Ad-E7 + IgG 2a, TC-1 + Ad-E7 + PD-1). Scale bars, 125 μm. (**B**) The H&E staining of tumor tissues in four groups (TC-1 + Ad-control + IgG 2b, TC-1 + Ad-control + PD-L1, TC-1 + Ad-E7 + IgG 2b, TC-1 + Ad-E7 + PD-L1). Red arrows indicate capillary, and black arrows indicate tumor capsule. Scale bars, 125 μm.

The H&E staining of tumor tissues of mice showed that, compared with the TC-1 + Ad-Contorl + IgG 2a group and TC-1 + Ad-Contorl + IgG 2b group, no significant differences in the thickness of tumor capsule were observed in monotherapy with PD-1/PD-L1 antibody (TC-1 + Ad-control + PD-1, TC-1 + Ad-control + PD-L1) (Figure 6A,B). In contrast, the tumor capsule with the monotherapy of Ad-E7 (TC-1 + Ad-E7 + IgG 2a, TC-1 + Ad-E7 + IgG 2b) was significantly thicker than the control groups. Combination therapy (TC-1 + Ad-E7 + PD-1, TC-1 + Ad-E7 + PD-L1) obviously increased the thickness of the tumor capsule. In addition, there was no significant difference in capillary density between monotherapy of the PD-1/PD-L1 antibody and the control groups. The capillary density of monotherapy of Ad-E7 was lower than the control group, and combination therapy decreased the capillary density further.

## 3. Discussion

Cervical cancer is one of the leading causes of death for women worldwide. Although HPV prophylactic vaccines have been gradually popularized, there is still no efficient treatment except surgery, radiotherapy and chemotherapy for cervical cancer patients. Therefore, it is essential to research new treatment options.

Therapeutic vaccination is one of the promising strategies. In recent years, there has been steady progress in developing various therapeutic DNA vaccines against HPV-associated malignancies. They are highly attractive because of their ability to induce CTL responses to eliminate HPV-infected or transformed cells [35]. Some vaccines have demonstrated anti-tumor effects in animal models. Mice vaccinated with pcDNA3-CRT/E6 have shown inhibited growth of E6-expressing tumor cells [36]. Synthetic neoantigen DNA-based vaccines (SNDVs) efficiently drive CD8^+^ T cell responses [37].

HPV16 E7 induces genetic instability through the degradation of pRb and activation of E2F, resulting in the development of cancers [38]. The constitutive expression of the E7 oncoprotein in the pre-cancer and cancer lesions could be recognized by the immune system. Considering the carcinogenicity and heterogeneity of E7 expression in tumors, we designed an HPV16 E7 mutant in which pRb binding activity is lost, and constructed a new therapeutic DNA vaccine targeting HPV16 E7 and packaged in an adenovirus vector (Ad-E7) in this study. We aim to describe the possibility of Ad-E7 in generating effective anti-tumor immune responses. We showed that Ad-E7 not only inhibited tumor growth, but also increased the proportion of IFN-γ-secreting CD8^+^ T cells in the spleen and tumor-infiltrating lymphocytes. However, Ad-E7 did not reduce the proportion of two main immunosuppressive cells (Tregs and MDSCs).

HPV is known for its capacity to escape from the surveillance of the immune system and establish persistent infection. The immune systems of cervical cancer patients rarely reverse the development of neoplastic cells [39]. Improving the efficacy of immunotherapy is one of the important goals of cancer research. Immune checkpoint therapy has shown success in treating patients with several cancer types. It can reverse tumor-induced immune exhaustion and generate durable objective tumor responses in patients [40]. PD-L1 is widely expressed in human tumor cells, and PD-L1 interacts with PD-1 on the T cell surface, helping tumor cells evade immune attack [41]. Therefore, blocking the PD-1/PD-L1 pathway will contribute to the treatment of cancers. D-mannose promoted the ubiquitylation and degradation of PD-L1, and synergized with the PD-1 antibody to suppress the growth of triple-negative breast cancer cells [42]. In addition, the mice that received PD-1 and the immune checkpoint inhibitory receptor CTLA-4 antibody had notably increased tumor-free survival [43]. Combinations of HPV therapeutic vaccines and immune checkpoint inhibitors have been studied in mice. Heterologous vaccination of pBI-11 and TA-HPV combined with the PD-1 antibody elicited a strong anti-tumor response, but the latter had no discernible effect alone [44]. In comparison, the PD-1 blockade did not augment the HPV E6/E7 peptide vaccine efficacy, which is probably due to the differences in the T cells generated by vaccination at disparate positions [45]. Adenovirus vector-based vaccines have been widely used for infectious diseases [46]. Here, we showed that the therapeutic effects were enhanced when Ad-E7 was combined with the PD-1/PD-L1 antibody. Moreover, the proportion of Tregs and MDSCs decreased significantly.

Several cytokines might be secreted to promote tumorigenesis. The expression of CC21 in mouse melanoma leads to a tolerogenic tumor microenvironment [47]. CCL2 can promote the malignant growth and metastasis of human liver cancer [48]. There is evidence that TNF-α can be detected in biopsies from human cancers, such as breast cancer and ovarian and renal cancer [29]. TGF-β decreases the activity of CD8^+^ T cells, which might be one reason for the immune surveillance of tumor cells [49]. MMPs promote tumor invasion and metastasis [50,51,52]. We detected the expression of certain cytokines and showed that both Ad-E7 vaccination and combination therapy down-regulated most of the cytokines, further inhibiting tumor progression.

It is a pity that we did not explore the effect of Ad-E7 on the survival and inhibition of metastasis. Considering the organ specificity for TC-1 cells, which were derived from lung epithelial cells, it is possible that the tumors may metastasize to the lung. Compared with intratumoral injections, subcutaneous intraperitoneal vaccination would be more appropriate, it would be of interest to clarify the anti-tumor immune responses with such injection method. All these issues need to be further explored in future studies.

In this study, Ad-E7 inhibits TC-1-induced HPV-associated tumor growth in the mouse model. It was demonstrated that Ad-E7 co-administered with the PD-1/PD-L1 antibody significantly inhibited the growth of tumors, which might be attributed to stronger CTL responses. In addition, combination therapy significantly decreased the proportion of Tregs and MDSCs. The thickness of the tumor capsule was also increased. Therefore, Ad-E7 might be a promising therapeutic candidate vaccine for cervical cancer patients.

## 4. Materials and Methods

### 4.1. Cell Culture

TC-1 cells were donated by Professor Kongnan Zhao (The University of Queensland). The other cells were purchased from American Type Culture Collection (ATCC). TC-1 is a tumorigenic cell line derived from primary lung epithelial cells of C57BL/6 mice, which has been co-transformed with the HPV-16 E6 and E7 oncogenes and human c-Ha-ras oncogene [53]. The TC-1 cells were cultured in Dulbecco’s modified Eagle medium (DMEM) supplemented with 10% fetal bovine serum (FBS), 0.2 mg/mL G418, and 0.1 mg/mL hygromycin. Mouse fibroblast cell line PA317, human embryonic kidney cell line 293T, and HEK293 were cultured in DMEM plus 10% FBS. Cervical cancer cell line CasKi was maintained in RPMI 1640 containing 10% FBS. All cells were cultured in humidified incubator with 5% CO_2_ at 37 °C.

### 4.2. Mice

Six-week-old female C57BL/6 mice were subcutaneously injected into the left flanks with TC-1 cells, which were diluted in 100 μL PBS. When the tumor reached a volume of approximately 200–250 mm^3^, different treatments were given to each group (5 mice per group). Ad-E7 and the Ad-control were diluted with 100 μL PBS and injected into the tumor, 2 × 10^9^ PFU per mouse; PD-1/ PD-L1 antibody (BioxCell, West Lebanon, NH, USA) or corresponding isotypes Rat IgG2a/IgG2b (BioxCell, West Lebanon, NH, USA) were dissolved in 100 μL antibody diluent and injected intraperitoneally, 200 μg per mouse. The tumor-bearing mice were vaccinated at day 6 and day 15 (for booster immunization) and treated with PD-1/PD-L1 antibody every three days. Tumor growth was monitored, and volume was measured every 3 days with calipers. The tumor volume was calculated with the formula (L × W^2^)/2, where L is the length and W is the width of the tumor. On day 19, mice were euthanized when the tumor volume reached 1.5 cm^3^. The tumor tissue and spleen were separated to prepare lymphocyte suspension, which was stained and detected by flow cytometry. In a second set of experiments, tumor tissues were fixed in formalin, embedded in paraffin and stained with H&E. All experimental animals were purchased from the Experimental Animal Centre of Shandong University.

### 4.3. Western Blotting

Total cellular proteins were extracted using Cell Lysate RIPA lysis buffer (Beyotime Biotechnology, Shanghai, China) with phenylmethanesulfonyl fluoride (PMSF, Beyotime Biotechnology, Shanghai, China) and a protease inhibitor (BestBio, Shanghai, China) at a 100:1:1 ratio. Proteins were separated by 10% SDS-PAGE and transferred to the PVDF membranes (Millipore, Burlington, MA, USA). After blocking in 5% skimmed milk, the membranes were incubated with primary antibodies against E7 (1:500; sc-6981; SANTA CRUZ, CA, USA), GAPDH (1:1000; 10494-1-AP; Proteintech, Wuhan, China), and β-actin (1:1000; ab8226; Abcam, Waltham, MA, USA). The goat anti-mouse HRP-conjugated secondary antibody (ZB-2305) and goat anti-rabbit HRP-conjugated secondary antibody (ZB-2301) were purchased from ZSGB-BIO (Beijing, China). The proteins were visualized using the Immobilon Western Chemiluminescent HRP Substrate Kit (Millipore, Burlington, MA, USA).

### 4.4. Quantitative Reverse-Transcription PCR

TRIzol reagent (Invitrogen, Carlsbad, CA, USA) was used to lyse the cells, and chloroform was added to extract the cellular RNA. After measuring the RNA concentration, the reverse transcription kit HiScript^®^ Q Select RT SuperMix (Vazyme, Nanjing, China) for qPCR was used to reverse transcribe the RNA to cDNA. A HiScript II One Step RT-PCR SYBR Green Kit (Vazyme, Nanjing, China) was used to amplify the products and monitored using a CFX96 Touch Real-Time PCR detection system (Bio-Rad, Hercules, CA, USA). The sequences of PCR primers were as follows:

GAPDH-R: GCCTTCTCCATGGTGGTGAA.
GAPDH-F: GCACCGTCAAGGCTGAGAAC.
CCL2-R: GCATTAGCTTCAGATTTACGGGT.
CCL2-F: TAAAAACCTGGATCGGAACCAAA.
CCL3-R: CAACGATGAATTGGCGTGGAA.
CCL3-F: TGTACCATGACACTCTGCAAC.
CCL5-R: CGACTGCAAGATTGGAGCACT.
CCL5-F: TTTGCCTACCTCTCCCTCG.
CCL12-R: ATCCAGTATGGTCCTGAAGATCA.
CCL12-F: ATTTCCACACTTCTATGCCTCCT.
CCL19-R: TAGTGTGGTGAACACAACAGC.
CCL19-F: CCTGGGAACATCGTGAAAGC.
CCL20-R: GAGGAGGTTCACAGCCCTTTT.
CCL20-F: ACTGTTGCCTCTCGTACATACA.
CCL21-R: GGGATGGGACAGCCTAAACT.
CCL21-F: GTGATGGAGGGGGTCAGGA.
CXCL10-R: GGCTCGCAGGGATGATTTCAA.
CXCL10-F: CCAAGTGCTGCCGTCATTTTC.
IL-2-R: GTCCAAGTTCATCTTCTAGGCAC.
IL-2-F: TGAGCAGGATGGAGAATTACAGG.
IL-4-R: GCCGATGATCTCTCTCAAGTGAT.
IL-4-F: GGTCTCAACCCCCAGCTAGT.
IL-10-R: GCAGCTCTAGGAGCATGTGG.
IL-10-F: CTTACTGACTGGCATGAGGATCA.
MMP2-R: CTTCCGCATGGTCTCGATG.
MMP2-F: ACCTGAACACTTTCTATGGCTG.
MMP3-R: TGTCCATCGTTCATCATCGTCA.
MMP3-F: GGCCTGGAACAGTCTTGGC.
TNF-α-R: CGATCACCCCGAAGTTCAGTAG.
TNF-α-F: CAGGCGGTGCCTATGTCTC.
TGF-β-R: AATGGTACCGTCAGTGCTGGAAATA.
TGF-β-F: TGGCTCATGTTGCAGAGGCTA.

### 4.5. Construction of the Adenovirus Vector

The GV269 vector was purchased from Shanghai Genechem Co., Ltd. (Shanghai, China), which is an E1 minus E3 minus adenovirus serotype 5 vector. Since the E1 gene is essential for replication, the absence of E1 makes the adenovirus unable to replicate [46]. Digested GV269 vector and E7 (delD21-C24) were co-transfected with helper plasmid (pBHG) into HEK293 cells to obtain replication-deficient recombinant human adenovirus Ad-E7. An Adeno-X^TM^ Virus Purification Kit (Becton Dickinson, Franklin Lakes, NJ, USA) was used for purification. Viral titer was determined using an end-point-dilution assay.

### 4.6. Flow Cytometry

To obtain single-cell suspensions, tumor tissues and spleen were digested by 0.2% collagenase II (Worthington, Lakewood, NJ, USA) for 30 min at 37 °C. A Mouse Tumor-infiltrating lymphocyte Isolation Kit (Solarbio, Beijing, China) and Mouse Spleen lymphocyte Isolation Kit (Solarbio, Beijing, China) were used to obtain lymphocyte suspensions, which from spleen and tumor tissues were filtered with 74 μm filter screen and stained with related fluorochrome-conjugated monoclonal antibodies. Antibodies used included the FITC anti-mouse CD8b.2 antibody (1:20; Cat#140404; Biolegend, San Diego, CA, USA), mouse IFN-gamma APC-conjugated antibody (1:10; IC485A-100; R&D Systems, Minneapolis, MN, USA), PE/Cyanine7 anti-mouse CD279 (PD-1) antibody (1:50; Cat#135216; Biolegend), FITC anti-mouse CD4 antibody (1:50; Cat#130308; Biolegend), APC anti-mouse CD25 antibody (1:50; Cat#101910; Biolegend), PE anti-mouse CD274 (B7-H1, PD-L1) antibody (1:50; Cat#124308; Biolegend), FITC anti-mouse/human CD11b antibody (1:50; Cat#101206; Biolegend), and PE anti-mouse Ly-6G/Ly-6C (Gr-1) antibody (1:50; Cat#108408; Biolegend). Cell surface staining was conducted for 30 min at 4 °C. Intracellular staining was conducted using a fixation/permeabilization buffer kit (Elascience). After staining, a flow cytometer (Beckman, Miami, FL, USA) was used for detection. Data were analyzed using FlowJo 10 software (Becton Dickinson, Franklin Lakes, NJ, USA).

### 4.7. Statistical Analysis

The data were expressed as means ± standard deviation (SD). Statistical analysis was performed using SPSS 20.0 (IBM, Chicago, IL, USA). The significant differences between the two groups were assessed by a two-tailed Student’s *t* test; multiple-group comparisons were performed using One-Way-ANOVA. Values of *p* < 0.05 were considered statistically significant.

## Data Availability

The data that support this study are available within the article and supplementary material.

## Supplementary Materials

The following supporting information can be downloaded at: https://www.mdpi.com/article/10.3390/ijms242015469/s1.
