# Supplementary material for: A Therapeutic DNA Vaccine Targeting HPV16 E7 in Combination with Anti-PD-1/PD-L1 Enhanced Tumor Regression and Cytotoxic Immune Responses
Source: Int J Mol Sci. 2023 Oct 23;24(20):15469. doi: 10.3390/ijms242015469 (PMC10607554; doi:10.3390/ijms242015469)
Supplement: Supplementary file 1 [file ijms-24-15469-s001.zip › ijms-2598835-supplementary.pdf]

Supplementary Materials for

A Therapeutic DNA Vaccine Targeting HPV16 E7 in Combination with Anti-PD-1/PD-L1 Enhanced Tumor Regression and Cytotoxic Immune Responses

Xuechao Han *et al.*

***Corresponding author.** Email: zhangweifang@sdu.edu.cn (W-F. Z.)

This file includes:

Figures S1 to S2

A

1 ATGCATGGAGATACACCTACATTGCATGAATATATGTTAGATTTGCAACC 50
51 AGAGACAACT~~GATCTCTACTGT~~TATGAGCAATTAAATGACAGCTCAGAGG 100
101 AGGAGGATGAAATAGATGGTCCAGCTGGACAAGCAGAACCGGACAGAGCC 150
151 CATTACAATATTGTAACCTTTTGTTGCAAGTGTGACTCTACGCTTCGGTT 200
201 GTGCGTACAAAGCACACACGTAGACATTCGTACTTTGGAAGACCTGTTAA 250
251 TGGGCACACTAGGAATTGTGTGCCCCATCTGTTCTCAGAAACCATAA 297

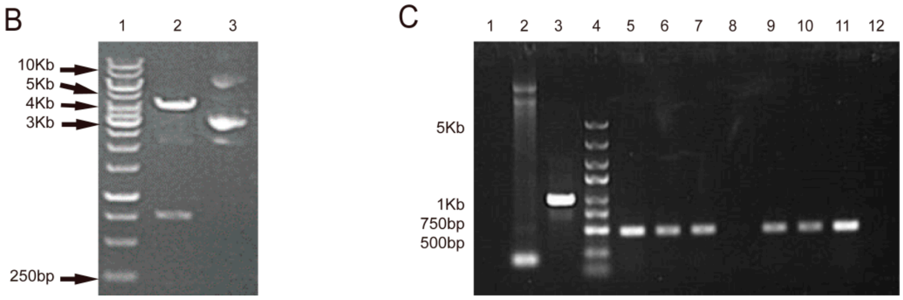

Figure S1. Construction of Ad-E7 vaccine.

(**A**) The DNA sequence of the HPV16 E7 (delD21-C24). (**B**) The enzyme cut assay of the GV269 vector (picture provided by Shanghai Genechem Co., Ltd.). Lane 1 is the DNA marker, which shows 10Kb, 8Kb, 6Kb, 5Kb, 4Kb, 3.5Kb, 3Kb, 2.5Kb, 2Kb, 1.5Kb, 1Kb, 750bp, 500bp and 250bp from top to bottom, lane 2 shows the enzyme-digested products, and lane 3 is the vector that was not digested by endonuclease. (**C**) The identification of positive transformants. Lane 1 is the negative control (ddH_2O), lane 2 is the negative control (empty vector), lane 3 is the positive control (GAPDH), lane 4 is the DNA marker, which is 5Kb, 3Kb, 2Kb, 1.5Kb, 1Kb, 750bp, 500bp, 250bp, 100bp from top to bottom, and lanes 5-12 are 8 transformants.

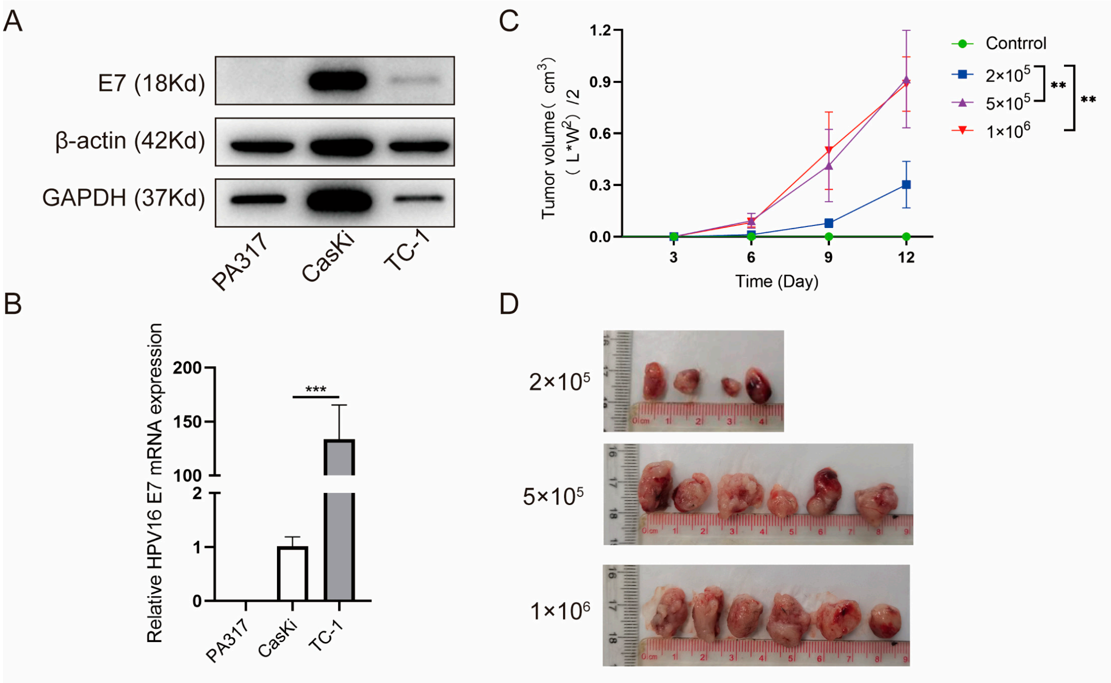

Figure S2. Identification of E7 expression and tumorigenic ability of TC-1 cells.

(**A**) Western blot was used to detect the protein expression of E7 in PA317 (HPV negative cells), CasKi (HPV positive cells) and TC-1 cells. (**B**) qRT-PCR was used to detect the mRNA level of E7 in PA317, CasKi and TC-1 cells. (**C**) The tumor growth curve of mice injected with TC-1 cells in three groups. (**D**) The tumors of the mice in three groups were isolated and photographed. $^{**}P<0.01$, $^{***}P<0.001$.
